# Evaluation of a National Breast Cancer Screening Program in a Middle-Income Country: The Case of Kazakhstan

**DOI:** 10.3390/ijerph23040532

**Published:** 2026-04-19

**Authors:** Yuliya Semenova, Zhandos Burkitbayev, Sanzhar Shalekenov, Oxana Shatkovskaya, Gauhar Dunenova, Alma Zhylkaidarova, Azat Chinaliyev, Baurzhan Anapiya, Asel Sadvakassova, Ayan Yerekesh, Zhadyra Karashutova, Almira Manatova, Lyudmila Pivina

**Affiliations:** 1 Department of Surgery, School of Medicine, Nazarbayev University, Astana 010000, Kazakhstan; yuliya.semenova@nu.edu.kz; 2 Management Board, National Research Oncology Center, Astana 010000, Kazakhstan; s.shalekenov@gmail.com (S.S.); 1972arty@mail.ru (O.S.); medicinaastana@gmail.com (A.C.); 3 Khoja Akhmet Yassawi International Kazakh-Turkish University, Turkestan 161200, Kazakhstan; gauhar.dunenova@gmail.com; 4 Specialized Consultative and Diagnostic Department, Almaty Regional Multispecialty Medical Center, Almaty 050060, Kazakhstan; almazhylkaidarova@gmail.com; 5 Vascular and Reconstructive Surgery Center, National Research Oncology Center, Astana 010000, Kazakhstan; plastic.nroc@gmail.com (B.A.); ayerekesh@gmail.com (A.Y.); 6 Women’s Health Center, National Research Oncology Center, Astana 010000, Kazakhstan; asadvakassova@outlook.com; 7 Department of Medical and Statistical Analysis, Salidat Kairbekova National Research Center for Health Development, Astana Z00T6E0, Kazakhstan; zh.karashutova@nrchd.kz; 8 Department of Scientific Management, National Research Oncology Center, Astana 020000, Kazakhstan; 9 Department of Emergency Medicine, Semey Medical University, Semey 071400, Kazakhstan; lyudmila.pivina@smu.edu.kz

**Keywords:** breast cancer, Kazakhstan, mammography, screening program

## Abstract

**Highlights:**

**Public health relevance—How does this work relate to a public health issue?**

- The study evaluates a national breast cancer screening program that affects millions of eligible women and represents a major public health investment.
- It addresses the gap between screening implementation and its population-level impact on breast cancer incidence and mortality in Kazakhstan.

**Public health significance—Why is this work of significance to public health?**

- Findings show that despite expanding coverage, the program has not produced observable changes in national breast cancer incidence or mortality trends.
- The results highlight critical discrepancies between program outputs, early detection performance and international benchmarks for effective screening.

**Public health implications—What are the key implications or messages for practitioners, policy makers and/or researchers in public health?**

- Suboptimal coverage levels and limited contribution of screening to early-stage breast cancer diagnoses suggest the need to refine program targeting and implementation.
- Policymakers may consider revising age eligibility, increasing participation and enhancing performance monitoring to ensure more efficient and impactful use of public health resources.

**Abstract:**

Breast cancer (BC) is the most common cancer in Kazakhstan, and a population-based breast cancer screening program was introduced in 2008, initially targeting women aged 50–60 years. It was subsequently expanded in 2018 to include women aged 40–70 years. This study evaluates the national BC screening program from its introduction in 2008 onward, focusing on program outputs, outcomes, and associated expenditures. Several administrative datasets and official sources were analyzed, including the legislative acts database, analytical reports on cancer screening programs, and cancer reporting forms. Trends in key indicators were summarized using the average annual percent change (AAPC). From the program’s inception, the absolute number of women screened increased steadily, with an AAPC of 6.23%. In contrast, the proportion of eligible women covered by screening declined over time, particularly following the expansion of the target age groups in 2018. Stage I BC detected through screening accounted for only about 50% of all stage I BC cases diagnosed nationwide, while the contribution of screening to stage II cancers was below 30%. Neither the introduction nor the subsequent expansion of the BC screening program was associated with statistically significant immediate or trend changes in national BC incidence or mortality rates. These findings may inform evidence-based discussions on potential refinements to BC screening policy and practice in Kazakhstan.

## 1. Introduction

Breast cancer is the most commonly diagnosed cancer worldwide. Its incidence and prevalence have increased substantially over recent decades and are projected to continue rising in the coming years [1,2,3]. Population growth and population aging are the primary drivers of this increasing burden, while genetic, environmental, and lifestyle factors also contribute [4]. The impact of breast cancer is unevenly distributed across countries, with middle-income settings experiencing a particularly high and growing burden. A range of strategies has been proposed to address this challenge, among which breast cancer screening remains one of the central approaches to disease control [1].

Several international organizations have developed breast cancer screening guidelines that differ in recommended screening intervals and target age groups. The World Health Organization (WHO) recommends biennial screening for apparently healthy women aged 50–69 years [5]. The American Cancer Society advises annual screening for women at average risk aged 45–54 years, with a transition to biennial screening from the age of 55 years onward [6]. In contrast, the U.S. Preventive Services Task Force recommends biennial screening for women at average risk starting at age 40 and continuing through age 74 years [7]. Differences in recommended age ranges and screening periodicity are largely guided by variations in the balance between anticipated benefits and potential harms of screening, as well as by differences in healthcare system capacities and population risk profiles [4].

Kazakhstan is an upper-middle-income country in Central Asia. It gained independence in 1991 following the dissolution of the Soviet Union, inheriting the Semashko model, a centrally planned, state-funded system that provided universal access to care, with a strong emphasis on hospital-based services. Since then, the country’s healthcare system has undergone substantial reform, including the introduction of market-oriented mechanisms and greater decentralization [8]. According to the latest GBD estimates, breast cancer incidence in Kazakhstan is similar to that in other Central Asian countries and lower than in Western Europe or North America; however, mortality is higher than in Western Europe and North America due to later-stage diagnosis, gaps in treatment access, and limited screening coverage [3].

A population-based breast cancer screening program was introduced in Kazakhstan in 2008, initially targeting women aged 50–60 years, and was subsequently expanded in 2018 to include women aged 40–70 years [9]. Although several studies have provided comprehensive evaluations of other publicly funded healthcare services in the country [10,11], research specifically focused on the breast cancer screening program remains limited. Existing publications have primarily examined breast cancer epidemiological trends during the period of screening implementation [12,13] or addressed selected technical aspects of program delivery [14,15]. Consequently, important dimensions of program performance have not been systematically assessed.

This study seeks to address this gap by examining the outputs (screening coverage), outcomes (cancers detected), and outlays (associated costs) of the national breast cancer screening program from its introduction in 2008 onward. In addition, the study evaluates the impact of the screening program on breast cancer incidence and mortality using a quasi-experimental design. The findings are intended to inform evidence-based discussion on potential improvements to breast cancer screening policy and practice in Kazakhstan.

## 2. Materials and Methods

### 2.1. Study Design

This was a retrospective observational study based on the analysis of multiple administrative datasets and official databases. The Adilet legal database maintained by the Institute of Legislation and Legal Information serves as a centralized repository of legal and regulatory acts, providing comprehensive access to current and historical legislation adopted in Kazakhstan [16]. It was consulted to obtain information on the structure and regulatory framework of the national breast cancer screening program, as well as tariff schedules for screening and diagnostic procedures related to its implementation. Data on screening performance indicators were extracted from analytical reports issued by the Kazakh Oncology Institute. Information on the annual number of newly diagnosed breast cancer cases, including stage distribution, and the number of deaths attributable to breast cancer, was obtained from the Ministry of Health (MoH) accounting and reporting forms No. 7 and No. 35, compiled by the MoH of Kazakhstan. Mid-year female population estimates used as denominators for rate calculations were retrieved from annual demographic yearbooks published by the Bureau of National Statistics of the Agency for Strategic Planning and Reforms [17].

### 2.2. Analysis of Policy Documents Regulating the Provision of Breast Cancer Screening Programs

A series of structured searches was conducted in the Adilet legal database by two researchers independently (Y.S. and A.M.) to identify official documents regulating the national breast cancer screening program in Kazakhstan. In cases of disagreement regarding whether a document should be included in the analysis, the opinion of a more experienced researcher (L.P.) was sought.

Searches were performed in both Kazakh and Russian, the languages used in legal and regulatory documents. In Russian, the following search terms were applied: (i) “рак мoлoчнoй железы” (breast cancer), (ii) “скрининг” (screening), (iii) “раннее выявление” (early detection), and (iv) “целевые группы населения” (target population groups). In Kazakh, the corresponding search terms were (i) “сүт безі қатерлі ісігі” (breast cancer), (ii) “скрининг” (screening), (iii) “ерте анықтау” (early detection), and (iv) “мақсатты пoпуляциялар” (target population groups).

Titles of all identified legal documents were screened, and a list of potentially relevant records was compiled. After removal of duplicates, full-text versions of the documents were retrieved and reviewed. Two MoH orders were identified as relevant; these regulated the initial breast cancer screening program for women aged 50–60 years and its subsequent expansion to women aged 40–70 years [18,19]. Information on the organization and implementation of the breast cancer screening program was extracted using standardized data extraction forms and summarized in tabular format.

### 2.3. Study Variables

Variables derived from the analytical reports on the breast cancer screening program included the annual number of women screened; the number of women diagnosed with ICD-10 codes C50 (malignant neoplasm of the breast), D24 (benign neoplasm of the breast), and N60 (benign mammary dysplasia); the number of women with these diagnoses who were followed up; and the stage distribution of malignant neoplasms. All indicators were available for the entire study period starting in 2008, with the exception of stage disaggregation, which was reported from 2011 onward.

Variables extracted from MoH reporting forms No. 7 and No. 35 included the annual number of newly diagnosed breast cancer cases; the number of women diagnosed with stage I disease, stage II disease, and stages I and II combined; and the annual number of deaths attributable to breast cancer. Data availability varied by year: information on newly diagnosed breast cancer cases was available from 1995 onward; data on stages I and II combined from 1997; and disaggregated data for stages I and II separately from 2017. Mortality data were available from 2002 onward. Mid-year female population estimates for Kazakhstan, used to calculate incidence and mortality rates, were obtained for the period starting in 1995.

### 2.4. Pharmacoeconomic Analysis

Direct expenditures related to breast cancer screening and subsequent diagnostic procedures were estimated from the healthcare provider’s perspective. Tariffs for healthcare services were obtained from MoH orders approving reimbursement rates for screening and diagnostic procedures; these tariffs are publicly available and were consistently reported from 2018 onward [20]. Annual tariffs for primary screening (two-view mammography and second radiologist interpretation) were extracted for each year and multiplied by the corresponding number of women screened. For additional diagnostics among women with screen-detected breast lesions, annual tariffs for breast ultrasound, targeted mammography, biopsy under local infiltration anesthesia, histopathological examination, and oncologist consultation were extracted and multiplied by the number of women undergoing these procedures.

The overall expenditure on the provision of breast cancer screening each year and subsequent diagnostic tests for lesions detected at screening were compared with the domestic general government health expenditure. For this, the WHO health expenditure database was consulted, and the corresponding values were extracted for each year beginning from 2018 onward [21]. The shares of expenditure on breast cancer screening, subsequent diagnostic tests, and breast cancer screening with diagnostic tests combined were presented as percentages of the domestic general government health expenditure.

### 2.5. Data Analysis

All statistical analyses, including graphical construction, were performed using SPSS software, version 24.0 (licensed to Nazarbayev University, Kazakhstan). Variables extracted for analysis were initially organized in Microsoft Excel spreadsheets, and the final datasets are provided in the Supplementary Materials. Temporal trends in each time series were assessed by calculating the average annual percent change (AAPC) with corresponding 95% confidence intervals (95% CI).

To evaluate the effects of the introduction of breast cancer screening in 2008 and its subsequent expansion in 2018 on breast cancer incidence and mortality, interrupted time-series (ITS) analyses were conducted using autoregressive integrated moving average (ARIMA) models. For each outcome indicator, the optimal underlying time-series model was first identified using the Expert Modeler function in SPSS. Within the ITS framework, both level (step) and slope (ramp) intervention functions were specified to capture the immediate and gradual effects of the policy changes introduced in 2008 and 2018. Model characteristics, including model type and stationary R^2^, were documented and presented in the results tables together with parameter estimates and their 95% confidence intervals for both level and trend changes.

Statistical significance was assessed using a two-sided alpha level of 0.05.

### 2.6. Ethics Statement

This study protocol was reviewed by the Ethics Committee of the National Research Oncology Center and was granted exempt status from ethics oversight due to the use of aggregated administrative data. Protocol №27 dated 24 April 2024.

## 3. Results

### 3.1. Performance of the Breast Cancer Screening Program: Output, Outcomes, and Outlay

A review of legal databases identified two MoH orders regulating the provision of breast cancer screening; the key elements of these orders are summarized in Table 1. Both documents stipulate that screening is conducted every two years and that nurses at primary healthcare facilities refer women to city or district polyclinics (in rural areas) equipped with the necessary imaging facilities. Mammography images are interpreted independently by two radiologists: the first reading is performed by a radiologist at the polyclinic where the screening is conducted, and the second by a radiologist at the regional oncology center, where all images are archived.

Overall, between 2008 and 2025, the absolute number of women screened for breast cancer increased steadily, with an average annual growth rate of 6.23%. In contrast, the proportion of eligible women covered by screening declined over time, largely reflecting the expansion of the target age groups in 2018. The number of breast lesions detected through screening increased at an average annual rate of 16.85%; however, the vast majority of detected lesions were benign. Most women with screen-detected lesions underwent follow-up, although a declining annual trend in follow-up was observed (AAPC = −3.19%) (Table 2).

Table 3 presents the stage distribution of breast cancers detected through screening; these data were available from 2011 onward. Between 2011 and 2019, stage II cancers were the most frequently detected, whereas from 2020 onward, stage I cancers became predominant. Overall, the detection of stage I cancers showed an increasing trend (AAPC = 8.01%), while the detection of stage II cancers declined (AAPC = −3.76%). During the early years of observation (2011–2013), stage III cancers were not uncommon among screen-detected cases; however, from 2014 onward, their proportion decreased, resulting in a consistently negative overall trend across the study period (AAPC = −15.59%). Stage IV cancers were also identified through screening, although they accounted for only a small proportion of cases.

Table 4 compares the detection of early-stage (I–II) breast cancers through screening with all stage I–II breast cancer cases diagnosed nationwide during the same period. Because the disaggregation of early-stage cancers into stages I and II for cancers detected outside the screening program was only reported from 2017 onward, the analysis is limited to the period 2017–2025. Overall, screening accounted for approximately half of all stage I cancers and for less than one third of stage II cancers, with both proportions showing a modest upward trend over time (AAPC 3.04% and 1.15%, respectively). Depending on the year, 60.92–71.58% of stage I–II cancers were diagnosed outside the screening program, namely among women in age groups not covered by mammography, through medical examinations or self-referral.

The direct costs associated with the provision of the breast cancer screening program are presented in Table 5. Between 2018 and 2025, both the cost per screened woman and the total cost of screening increased, reflecting tariff adjustments in response to national currency inflation. The share of direct screening costs within Domestic General Government Health Expenditure ranged from 0.16 to 0.26%, while expenditures on additional diagnostic tests ranged from 0.21 to 0.35%. Combined, direct screening costs and the costs of additional tests accounted for 0.39–0.54% of Domestic General Government Health Expenditure.

### 3.2. Impact of the Breast Cancer Screening Program on Nationwide Breast Cancer Incidence and Mortality

**Figure 1:**
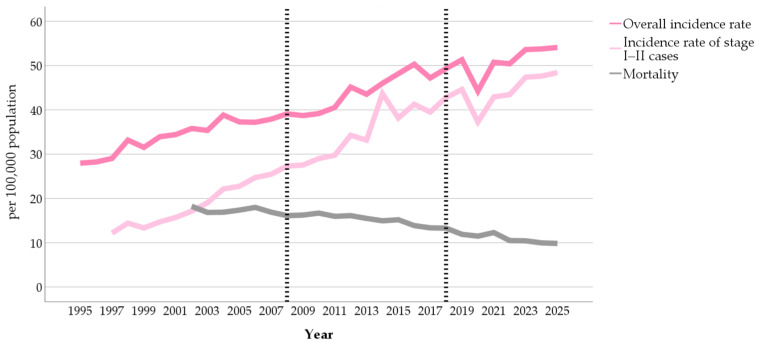
Temporal trends in breast cancer incidence and mortality (vertical dashed lines indicate the introduction of the breast cancer screening program in 2008 and its expansion in 2018).

Nationwide, the incidence rate of breast cancer increased from 27.97 to 54.08 per 100,000 female population between 1995 and 2025 (AAPC = 2.14, 95% CI: 1.94–2.34). The incidence of stage I–II breast cancer rose more sharply, from 12.18 per 100,000 in 1997 to 48.43 per 100,000 in 2025 (AAPC = 5.05, 95% CI: 4.49–5.61). The proportion of stage I–II cancers among all breast cancer cases also increased, from 41.94% in 1997 to 89.53% in 2025 (AAPC = 2.95, 95% CI: 2.49–3.42). In contrast, the mortality rate declined steadily from 18.23 per 100,000 in 2002 to 9.84 per 100,000 in 2025 (AAPC = −2.63, 95% CI: −3.01 to −2.25) (Figure 1).

Table 6 presents the estimated effects of the introduction of the breast cancer screening program in 2008 and its expansion in 2018 on incidence and mortality rates. For overall incidence, neither intervention was associated with a significant immediate change in level or in post-intervention trend. The 2008 intervention corresponded to a level change of −1.82 cases per 100,000 population (SE 2.11; *p* = 0.399) and a trend change of +0.15 cases per 100,000 per year (SE 0.19; *p* = 0.901). The 2018 intervention showed an estimated level change of −3.86 cases per 100,000 (SE 3.17; *p* = 0.238) and a trend change of −0.61 per year (SE 2.75; *p* = 0.826). For stage I–II cancers, neither intervention resulted in a clear immediate or sustained change. The 2008 intervention was associated with an estimated level change of 0.48 cases per 100,000 and a trend change of 0.03 per year, while the 2018 intervention showed an estimated level change of 2.46 cases per 100,000 and a trend change of −0.55 per year. The negative trends observed post-intervention for both overall and stage I–II incidence likely reflect the decline in 2020, coinciding with the onset of the COVID-19 pandemic.

For mortality, neither intervention was associated with a significant immediate change or trend change. The 2008 intervention corresponded to an estimated level change of −0.47 deaths per 100,000 (SE 0.81; *p* = 0.570) and a trend change of −0.04 per year (SE 0.43; *p* = 0.928). The 2018 intervention showed an estimated level change of 0.42 deaths per 100,000 (SE 0.82; *p* = 0.614) and a trend change of −0.19 per year (SE 0.39; *p* = 0.639).

## 4. Discussion

This study provides a comprehensive evaluation of the nationwide breast cancer screening program from its inception to the present, with the aim of identifying lessons relevant for potential policy refinement. The number of women screened for breast cancer increased steadily, although the proportion of eligible women covered declined, largely due to the expansion of target age groups in 2018. The number of lesions detected through screening also rose, with the majority being benign. In the early years of the program, stage II cancers were most commonly detected, whereas from 2020 onward, stage I cancers became predominant, showing an overall increasing trend. Despite this, a substantial share of early-stage cancers continued to be diagnosed outside the screening program. The costs of the screening program, including additional diagnostic tests, increased with inflation and together accounted for approximately 0.4–0.5% of Domestic General Government Health Expenditure. Overall, neither the introduction nor the expansion of the program in 2008 and 2018 was associated with clear changes in breast cancer incidence or mortality trends, suggesting that observed fluctuations in rates likely reflect other factors. These findings need contextual interpretation.

### 4.1. Cost-Effectiveness of Expanded Breast Cancer Screening Program

A previous study evaluating the cost-effectiveness of mammography in Kazakhstan prior to the 2018 expansion of the screening program concluded that it was cost-effective, resulting in savings in total treatment costs per screened cohort compared with an unscreened cohort, amounting to USD 2,708,953, and generating 818 quality-adjusted life years (QALYs) [22]. The present study adopts a different pharmacoeconomic perspective, comparing the total health expenditure for breast cancer screening with the Domestic General Government Health Expenditure. On one hand, the resulting proportion (0.4–0.5%) appears modest and reasonable, as preventive care, including screening programs, accounts for approximately 3% of total health expenditure in Organisation for Economic Co-operation and Development (OECD) countries [23]. On the other hand, six additional conditions affecting adults are currently targeted by population-based screening in Kazakhstan, including cervical [24] and colorectal cancer [25], arterial hypertension and ischemic heart disease [26], diabetes [27], and glaucoma [28]. This suggests that cumulative expenditures on preventive programs may exceed the 3% typically observed in OECD countries, raising questions about the long-term sustainability of Kazakhstan’s preventive health strategies.

### 4.2. Outputs and Outcomes of Breast Cancer Screening Program

Another important aspect of the breast cancer screening program relates to its output. The number of women screened increased steadily over time, with the most pronounced rise observed in 2018 following the expansion of eligible age groups; compared with 2017, the number of women screened increased by 56%. However, this expansion was accompanied by a proportional decline in coverage, and after 2018, the mean screening coverage was 52.6%. WHO guidance indicates that high screening coverage is necessary to achieve population-level impact, commonly interpreted as at least 70% of the target population [29]. European Commission and European Union screening guidelines specify that coverage of ≥70% is required for program effectiveness, with ≥75% considered optimal [30]. Although no formal national coverage target is defined in the United States, uptake levels of ≥70% are frequently cited in program evaluations and public health literature as desirable benchmarks [31]. From this perspective, the coverage levels observed in the Kazakhstani breast cancer screening program after its expansion appear suboptimal and may constrain its potential population-level effectiveness.

Several indicators collected in this study can inform the evaluation of breast cancer screening program outcomes. The screen-detected cancer rate observed ranged from 0.06% to 0.24% and showed a modest increase following the 2018 expansion of eligible age groups. Internationally, organized screening programs report a range of detection rates: some programs achieve 0.4–0.9% [32,33,34], while others report lower rates of 0.1–0.3% [35,36]. Countries with higher breast cancer incidence generally report higher screen-detected cancer rates [37]. In addition, detection rates tend to be higher during initial screening rounds, which capture prevalent cancers, whereas subsequent screens typically detect fewer cases, reflecting incident cancers in populations with established coverage [38]. Overall, low detection rates may indicate lower program sensitivity, a lower-risk screened population, or both [39].

### 4.3. Breast Cancer Incidence and Risk Factors

In Kazakhstan, the incidence of breast cancer is relatively low compared with high-income countries, as indicated by both the findings of this study and GLOBOCAN estimates [40]. This may partly explain the low rate of screen-detected cancers. In addition, as of 2024, the proportion of the population aged 65 years and older is approximately 8.65% [41], which is below the global average (approximately 10%) [42]. Although this share is increasing and is projected to exceed 14% by 2050 [43], population ageing in Kazakhstan remains less advanced than in many high-income settings. Aside from age, several other factors may help explain the relatively lower incidence of breast cancer in Kazakhstan. Prolonged breastfeeding is a well-known protective factor [44], and a case–control study in Kazakhstan reported that breastfeeding for 6–24 months was associated with a reduced risk of breast cancer [45]. Higher parity has also been consistently associated with a lower risk of breast cancer [46], and in Kazakhstan, the total fertility rate remains relatively high, with women having, on average, around three children [47], which may contribute to the observed epidemiological patterns.

Taken together, the relatively low incidence of breast cancer and the smaller proportion of older individuals do not, on their own, provide a strong epidemiological rationale for expanding the screening program to include both younger and older age groups. Such expansions are more commonly considered in contexts with higher disease incidence or a larger and more rapidly ageing population [48]. Therefore, the expansion of the breast cancer screening program is likely driven more by policy and health system priorities, such as increasing coverage, aligning with international practices, and addressing stakeholder or public expectations, than by shifts in the underlying epidemiological profile alone.

Although Kazakhstan has made substantial investments to improve the quality of its breast cancer screening program (through the introduction of digital mammography in all screening clinics and the training and qualification upgrading of physicians [49]), the low screen-detected cancer rate, combined with relatively low incidence and proportion of older population, highlights the need to reassess program targeting in order to ensure that the screening strategy delivers meaningful population-level benefits.

### 4.4. Overall Efficacy of Breast Cancer Screening Program

The proportion of early-stage cancers detected through screening is a widely used indicator of program effectiveness. In mature screening programs, a growing share of screen-detected cancers is typically diagnosed at stage I, and stage I and II cancers together generally account for the majority of cancers identified through screening [50]. In this study, however, stage I breast cancers detected through screening represented only about 50% of all stage I breast cancers diagnosed nationwide, while the contribution of screening to stage II cancers was even lower (less than 30%). Overall, stage I and II breast cancers identified through screening accounted for less than 40% of all cancers diagnosed at these stages nationally, which is below international benchmarks. For comparison, organized mammography in Sweden detects approximately 60% of all breast cancers [51], while in the UK, over half of all diagnosed breast cancers are identified through the national screening program [52]. Similar patterns have been reported in Canada, where screening accounts for the majority of new breast cancer diagnoses in the population offered screening [53].

The lack of observable effects following the introduction of the screening program in 2008 and its expansion in 2018 on overall breast cancer incidence (including stage I–II cancers) and on breast cancer mortality aligns with the findings of suboptimal coverage and the low contribution of screen-detected early-stage cancers to overall early-stage incidence. Taken together, these findings indicate that, in its current configuration, the screening program contributes only marginally to improvements in breast health at the population level. While Kazakhstan has made substantial efforts to establish and sustain a national screening program, the observed decline in breast cancer mortality may also reflect the Ministry of Health’s broader investments in cancer care, including the introduction and wider availability of advanced diagnostic and therapeutic modalities [54], alongside screening activities. Still, significant challenges remain, as late-stage breast cancers continue to present with poor survival outcomes [55].

The observation that many breast cancer cases are identified outside the screening program may indicate the presence of barriers to screening. A recent study conducted in Eastern Kazakhstan found that lack of awareness of the benefits of breast cancer screening, as well as competing work and household responsibilities, are among the key barriers to screening uptake [56]. Priority areas for improvement include enhancing the current screening program, potentially by narrowing age eligibility and increasing coverage rates, while addressing the barriers faced by Kazakhstani women in accessing screening and tackling issues of inequity in service provision.

### 4.5. Study Strengths and Limitations

This study benefits from the use of a large administrative dataset, which allowed for a comprehensive assessment of the nationwide breast cancer screening program from the time it was introduced. The use of a quasi-experimental design (interrupted time series analysis) represents another strength, as relatively few studies in Kazakhstan have applied this methodology to evaluate the impact of health policies [57]. In addition, the integration of quantitative and qualitative analyses further strengthens the study.

At the same time, several limitations should be acknowledged. Most importantly, the aggregated nature of the data does not permit analyses at the individual level, limiting the ability to examine causal relationships or to clearly separate program-related effects from broader population trends. As such, it is not possible to examine the characteristics of women who received care or to assess individual-level factors that may contribute to or protect against breast cancer. The rationale behind the decision to expand the breast cancer screening program to include younger and older age groups could also not be explored using the available dataset. In addition, some key indicators were available only for a relatively short period, often starting well after the screening program had already been implemented. This limits the evaluation of long-term patterns and makes it difficult to assess program performance during its initial phases, including baseline conditions and early changes over time. Finally, a number of indicators that are central to the evaluation of screening quality, such as interval cancer rates, recall rates, and false-positive and false-negative rates, were not available, restricting a more detailed assessment of diagnostic performance.

## 5. Conclusions

This study offers a long-term, system-level assessment of the nationwide breast cancer screening program in a middle-income country, Kazakhstan, and identifies important gaps between program design, implementation, and population-level impact. The findings point to the need for a structured dialogue on potential program refinement to ensure closer alignment with the epidemiological profile and needs of the target population. Priority areas may include revisiting age eligibility criteria in relation to age-specific incidence patterns, improving participation and coverage among eligible women, and strengthening program monitoring through the routine collection of key quality indicators. Together, these measures could enhance the responsiveness and effectiveness of the screening program while also promoting a more efficient use of public resources.

## Figures and Tables

**Table 1:** Legislative provisions regulating breast cancer screening program in Kazakhstan.

Year of Issuance [Ref.]	Target Population	Screening Periodicity	Screening Setting	Screening Method	Additional Diagnostic Tests
2008 [15]	Women aged 50–60 years not registered for BC *	Every two years	City/district polyclinic/mobile screening unit	Two-view mammography with images read independently by two radiologists; interpretation is carried out in accordance with BI-RADS **	Not specified
2018 [17]	Women aged 40–70 years not registered for BC *				-Breast ultrasound -Targeted mammography -Oncologist consultation -Gynecologist consultation

BC *—breast cancer. BI-RADS **—Breast Imaging-Reporting and Data System.

**Table 2:** Annual breast cancer screening coverage and diagnostic outcomes.

Year	Number of Women Screened	Coverage (%)	Number of Detected Lesions	Proportion of Benign Lesions (%)	Screen-Detected Cancer Rate (%)	Proportion of Patients with Detected Lesions Who Received Follow-Up (%)
2008	410,438	N/R **	N/R	N/R	0.09	N/R
2009	409,918	N/R	N/R	N/R	0.09	N/R
2010	428,498	N/R	N/R	N/R	0.06	N/R
2011	455,745	77.7	11,832	95.72	0.11	69.37
2012	459,816	78.4	49,492	98.95	0.11	89.15
2013	379,903	64.8	45,695	98.84	0.14	93.57
2014	386,112	64	45,077	98.59	0.16	96.40
2015	408,824	67.1	96,702	99.29	0.17	87.94
2016	389,352	62.3	100,689	99.18	0.21	85.30
2017	420,560	65.6	105,962	99.23	0.19	78.46
2018	754,465	48.7	172,676	99.12	0.20	55.11
2019	870,202	54.8	165,230	98.75	0.24	64.88
2020	744,972	45.8	150,371	99.37	0.13	48.82
2021	787,619	47.5	186,334	99.26	0.18	46.09
2022	808,503	47.9	250,517	99.41	0.18	38.19
2023	918,464	54.9	172,123	98.74	0.24	68.90
2024	937,205	61.9	168,199	98.88	0.20	76.37
2025	959,830	66.4	157,895	98.84	0.19	72.77
AAPC (95% CI) *	6.23 (4.29; 8.22)	−2.24 (−4.06; −0.38)	16.85 (10.47; 23.60)	0.09 (−0.02; 0.20)	5.78 (3.30; 8.31)	−3.19 (−6.23; −0.04)

AAPC (95% CI) *—annual percent change with 95% confidence interval. N/R **—not reported.

**Table 3:** Stage distribution of breast cancer cases detected through screening.

Year	Number of Breast Cancer Cases	Stage I		Stage II		Stage III		Stage IV	
		N	%	N	%	N	%	N	%
2011	469	99	21.11	304	64.82	55	11.73	11	2.35
2012	633	141	22.27	421	66.51	66	10.43	5	0.79
2013	616	132	21.43	390	63.31	88	14.29	6	0.97
2014	700	201	28.71	438	62.57	54	7.71	7	1.00
2015	765	230	30.07	490	64.05	40	5.23	5	0.65
2016	895	350	39.11	496	55.42	41	4.58	8	0.89
2017	886	328	37.02	520	58.69	32	3.61	6	0.68
2018	1625	738	45.42	813	50.03	56	3.45	18	1.11
2019	1752	796	45.43	867	49.49	64	3.65	25	1.43
2020	1072	521	48.60	502	46.83	38	3.54	11	1.03
2021	1402	671	47.86	668	47.65	52	3.71	11	0.78
2022	1570	788	50.19	722	45.99	46	2.93	14	0.89
2023	1875	1009	53.81	840	44.80	15	0.80	11	0.59
2024	1922	1085	56.45	805	41.88	28	1.46	4	0.21
2025	1836	1050	57.20	771	42.00	11	0.60	4	0.21
AAPC (95% CI) *	10.41 (7.82; 13.05%)	19.24 (15.41; 23.21)	8.01 (6.52; 9.51)	6.36 (3.94; 8.83)	−3.76 (−4.18; −3.15)	−6.80 (−10.85; −2.57)	−15.59 (−19.02; −12.01)	0.65 (−6.48; 8.32)	−8.83 (−14.31; −3.01)

AAPC (95% CI) *—annual percent change with 95% confidence interval.

**Table 4:** Detection of early-stage breast cancer through screening in relation to all national cases.

Year	Total Number of Stage I Cases Nationwide	Proportion of Stage I Cases Detected Through Screening (%)	Total Number of Stage II Cases Nationwide	Proportion of Stage II Cases Detected Through Screening (%)	Total Number of Stage I and II Cases Nationwide	Proportion of Stage I and II Cases Detected Through Screening (%)
2017	1001	32.77	2676	19.43	3677	23.06
2018	1441	51.21	2590	31.39	4031	38.48
2019	1553	51.26	2702	32.09	4255	39.08
2020	1261	41.32	2338	21.47	3599	28.42
2021	1472	45.58	2700	24.74	4172	32.09
2022	1686	46.74	2709	26.65	4395	34.36
2023	1944	51.90	2850	29.47	4794	38.57
2024	2081	52.14	2858	28.17	4939	38.27
2025	2144	48.97	2925	26.36	5069	35.92
AAPC (95% CI) *	8.49 (4.78; 12.33)	3.04 (−1.15; 7.41)	1.52 (−0.18; 3.25)	1.15 (−4.19; 6.78)	3.96 (1.93; 6.04)	3.06 (−2.08; 8.47)

AAPC (95% CI) *—annual percent change with 95% confidence interval.

**Table 5:** Direct costs associated with mammography screening and subsequent diagnostic tests.

Year	Mammography			Additional Tests			Mammography and Additional Tests as a Proportion of DGGHE (%)
	Cost per Screened Woman (NCU°)	Total Cost of Screenings (Million NCU)	Proportion of DGGHE” (%)	Cost per Woman Tested (NCU)	Total Cost of Additional Tests (Million NCU)	Proportion of DGGHE (%)	
2018	2637.40	1989.83	0.19	16,489.30	2847.31	0.27	0.46
2019	3248.40	2826.76	0.24	16,489.30	2724.53	0.23	0.48
2020	4215.50	3140.43	0.18	24,884.80	3741.95	0.21	0.39
2021	4456.00	3509.63	0.16	28,585.40	5326.43	0.25	0.41
2022	5479.00	4429.79	0.19	33,777.30	8461.79	0.35	0.54
2023	6860.60	6301.21	0.21	39,683.30	6830.41	0.23	0.44
2024	9571.50	9002.01	0.26	42,749.10	7896.14	0.23	0.50
2025	9972.70	9269.46	0.24	44,090.90	7866.52	0.21	0.45
AAPC (95% CI) *	21.53 (18.44; 24.70)	25.21 (20.41; 30.20)	3.23 (−2.83; 9.66)	17.04 (12.38; 21.88)	19.13 (10.13; 28.87)	1.36 (−7.75; 5.47)	0.82 (−3.32; 5.14)

AAPC (95% CI) *—annual percent change with 95% confidence interval. NCU°—national currency unit. DGGHE”—Domestic General Government Health Expenditure.

**Table 6:** Effect of the 2008 introduction and 2018 expansion of breast cancer screening program on incidence and mortality.

Tested Indicators		Model Parameters		Level Change		Trend Change	
		Model Type	Stationary R^2^, *p*-Value	Estimate (Standard Error)	*p*-Value	Estimate (Standard Error)	*p*-Value
Overall incidence	Introduction of breast cancer screening program in 2008	ARIMA (0.1.1)	0.588, *p* = 0.435	−1.82 (2.11)	0.399	0.15 (0.19)	0.901
	Expansion of breast cancer screening program in 2018			−3.86 (3.17)	0.238	−0.61 (2.75)	0.826
Incidence of stage I–II cancers	Introduction of breast cancer screening program in 2008	ARIMA (0.1.0)	0.021, *p* = 0.042	0.48 (3.64)	0.896	0.03 (1.59)	0.986
	Expansion of breast cancer screening program in 2018			2.46 (3.70)	0.512	−0.55 (1.74)	0.755
Mortality	Introduction of breast cancer screening program in 2008	ARIMA (0.1.0)	0.040, *p* = 0.477	−0.47 (0.81)	0.570	−0.04 (0.43)	0.928
	Expansion of breast cancer screening program in 2018			0.42 (0.82)	0.614	−0.19 (0.39)	0.639

## Data Availability

The raw data supporting the conclusions of this article will be made available by the authors, without undue reservation.

## Supplementary Materials

The following supporting information can be downloaded at https://www.mdpi.com/article/10.3390/ijerph23040532/s1: Table S1: Minimal dataset.

## Abbreviations

The following abbreviations are used in this manuscript:

AAPC	Average annual percent change
ARIMA	Autoregressive integrated moving average
BC	Breast cancer
BI-RADS	Breast Imaging-Reporting and Data System
CI	Confidence Interval
DGGHE	Domestic General Government Health Expenditure
ITS	Interrupted time-series
MoH	Ministry of Health
NCU	National Currency Unit
OECD	Organisation for Economic Co-operation and Development
SE	Standard error
QALYs	Quality-adjusted life years
WHO	World Health Organization
